# The Effect of a Single Bout Circuit Resistance Exercise on Homocysteine, hs-CRP and Fibrinogen in Sedentary Middle Aged Men

**Published:** 2011

**Authors:** Nahid Bizheh, Mohsen Jaafari

**Affiliations:** 1 *Faculty of Physical Education and Sport Sciences, Ferdowsi University of Mashhad, Iran*; 2 *Kish International Campus, University of Tehran, Iran*

**Keywords:** Atherosclerosis, C-reactive protein, Exercise, Fibrinogen, Homocysteine

## Abstract

**Objective(s):**

Homocysteine, hs-CRP and fibrinogen are three novel cardiovascular risk factors. The aim of this study was to examine the effect of a single bout of circuit resistance exercise on homocysteine, hs-CRP and fibrinogen levels in healthy and inactive men.

**Materials and Methods:**

The subjects were randomly divided into the experimental group (n= 14) and the control group (n= 9). Circuit training comprised ten exercises with 35% of one repetition maximum intensity. Blood samples were collected thirty minutes before and immediately after training. The data were analyzed using Kolmogorov Smirnov and independent samples T test in SPSS15.0 Software (*P*< 0.05 was regarded significant).

**Results:**

Analysis of data showed the significant elevation of serum homocysteine and hs-CRP levels after training in the experimental group but not in the control group. No significant changes of fibrinogen levels were observed in both groups.

**Conclusions:**

During the exercise, the elevation of creatine synthesis for ATP production increases homocysteine levels. Moreover, muscle-derived interleukin-6 (a stimulator of glycogenolysis in the liver) induces hepatic production of CRP. Pathological or beneficial consequences of these changes are not clearly specified. Furthermore, more research is needed to show the acute and chronic effects of physical activity on novel cardiovascular risk factors.

## Introduction

Cardiovascular diseases (CVDs), especially atherosclerosis, are the leading causes of mortality worldwide especially atherosclerosis. Traditional CVD risk factors include smoking, physical inactivity, high fat diet, hypertension and diabetes mellitus, but new studies have shown that these risk factors do not fully account for CVD (1). For example, plenty of persons who suffer from myocardial infarction have normal cholesterol levels (2). Homocysteine (Hcy), high sensitive C-reactive protein (hs-CRP) and fibrinogen are three novel cardiovascular risk factors that independently and strongly predict the risk of this disease (1). 

Hcy is a non-essential amino acid produced in the methionine metabolism. Genetic enzymatic defects lead to the elevation of Hcy levels (3). Hcy induces the LDL aggregation, forms foam cells and suppresses the proliferation of endothelial cells (4). C-reactive protein (CRP) is an acute phase protein involved in systemic inflammation. Lipid and arterial tissue synthesize CRP under induction of interleukin-6 (IL-6) and leptin. CRP induces expression of pro inflammatory cytokines, including inter cellular adhesion molecule-1 (ICAM-1), vascular cell adhesion molecule-1 (VCAM-1), tumor necrosis factor alpha (TNF-α), interleukin-1 (IL-1) and IL-6, activates the complement system, regulates the phagocytosis of LDL by macrophages and decreases the expression of nitric oxide synthase (5). Fibrinogen, a molecule produced in the liver, is consisted of a glycoprotein and three polypeptide chains (α, β, γ). Fibrinogen binds to GP1b and GP2b/3a receptors of platelets and stimulates adhesion and aggregation of platelets. It has a central role in the fibrin thrombus formation and increases plasma viscosity (6).

Glecek *et al* (2007) entered twenty two healthy and sedentary subjects in a one session aerobic exercise program on the treadmill to investigate the acute effect of aerobic exercise on Hcy level in plasma and reported the significant elevation of Hcy after exercise in these subjects (7). In a study, CRP increased after a forty two km marathon race. The subjects included seventy male and twenty female trained runners. It also significantly increased after 160 km triathlon in eighteen male trained athletes. Another study showed a meaningful increment in CRP levels after the 6-day ultra-marathon in eight trained runners (8). Tsao *et al* (2009) studied the effects of three exercise intensities (65%, 85% and 100% of VO_2_max) on serum levels of leptin and CRP. They noted that exercise intensity had no effect on the responses of leptin and CRP, because after all three intensity exercises, serum leptin level decreased and serum CRP level increased significantly (9). Lee-Saw-Hee *et al* (2001) reported a meaningful elevation of fibrinogen after treadmill exercise in patients with atrial fibrillation (10). 

There is not enough research about the acute effects of exercise and physical activity on levels of Hcy, CRP and fibrinogen (2,7). Also no research was found about the effects of a single bout of circuit resistance exercise on blood levels of these risk factors in healthy and inactive middle aged men. Understanding the mechanisms of changes in these inflammatory markers after one session of resistance exercise is essential for specifying the exact cardio protective benefits of long term exercise (11). Thus the aim of this study was to investigate the acute effect of one session circuit resistance exercise on Hcy, hs-CRP and fibrinogen in healthy and sedentary middle aged men who were inhabitants in Mashhad city, North-east of Iran. 

## Materials and Methods

This research plan was confirmed by Research Assembly of Physical Education and Sport Sciences Faculty of Ferdowsi University of Mashhad. The subjects of this study were twenty three healthy and inactive men who randomly assigned into the experimental (n= 14) and control (n= 9) groups. Before starting the program, written informed consents were taken from all subjects. The levels of health and physical activity of the subjects were determined using general practice physical activity questionnaire, physical activity readiness questionnaire and medical survey (including electrocardiogram and blood pressure tests) by a specialist physician. The subjects were nonsmokers, received no drugs and had no metabolic disease and physical impairment affecting their performance. Furthermore, the levels of aerobic power of the subjects were measured via treadmill (Techno Gym S.p.A, Class: RUNRACE 1400HC, ) and their body composition status specified via Body Composition Analyzer (InBody 720). Bruce's protocol was used for determination of each subject`s VO_2_max on the treadmill. A training session was held five days before the main session for acquaintance of subjects in the experimental group with resistance exercises and determination of their one repetition maximum (1RM) per exercise (maximum muscular strength (1RM) = [lifted weights] ÷ [1–{0.02×repetitions}]).

Training session was started at eight o`clock in the morning. Resistance training program included ten exercises: arm curl, triceps extension, back extension, sit-up, squat 90^o^, leg curl, bench press, overhead press, dead lift, and seated row. Duration of each exercise was twenty seconds and there were three circuits with one minute rest between each. The subjects performed each exercise with repetitions maximum within twenty seconds (12). 

The intensity of exercises was equivalent to 35% of one repetition maximum of the subjects. The subjects were asked to avoid any medication and intense physical activity three days before the main session. 

In the fasting state, blood samples were taken from left brachial vein thirty minutes before starting the training program and immediately after ending it. Then the blood samples were sent to the laboratory for analysis. All biochemical procedures were done via an auto analyzer system. Serum Hcy was measured using a special kit and Elisa method (Axis^®^ Homocysteine EIA). To determine the serum levels of hs-CRP, commercial kits of Pars Azmoon Company (Iran, Tehran) and immunoturbidimetric method were used (2). Furthermore, plasma levels of fibrinogen were determined using Takeda method and a special kit (13). 

## Results

The descriptive characteristics, the body composition status and initial levels of Hcy, hs-CRP and fibrinogen are shown in Table 1 and Table 2. In Table 3, the results of Kolmogorov Smirnov test are shown. This test showed the normality of the data. Before the onset of the exercise, there were no significant differences between groups in body composition variables including weight, body mass index (BMI), percent body fat (PBF), visceral fat area (VFA), and waist to hip ratio (WHR). Moreover, no significant differences were observed between groups in serum levels of Hcy, hs-CRP and plasma levels of fibrinogen. Independent samples T test showed a significant increment in serum levels of Hcy and hs-CRP after the exercise (*P*< 0.05), but plasma levels of fibrinogen did not significantly change.

**Table 1.** General characteristics of subjects and differences among them before exercise

Variables	Exercise group	Control group	*P* value
Age	44.93±4.14	43.11±5.16	0.10
Height (m)	1.72±0.05	1.76±0.04	0.42
Weight (kg)	82.28±9.93	83.90±10.95	0.13
BMI (kg/m^2^)	27.70±2.79	27.26±2.36	0.12
PBF (%)	26.38±4.43	28.20±5.40	0.48
WHR	0.93±0.03	0.94±0.03	0.67
VFA (cm^2^)	125.68±27.62	142.18±35.92	0.91
VO_2_max (l/min)	1.96±0.46	1.63±0.24	0.06

**Table 2.** Pre-test and post-test levels of dependent variables and differences between two groups. *P* values are related to independent samples t-test

Variable	Groups	Pre-test	Post-test	t	*P* value
Homocysteine (mmol/l)	Experimental group	7.88±0.7	8.45±0.79	2.12	0.04
	Control group	7.56±1.09	7.51±0.88		
hsCRP (mg/l)	Experimental group	1.98±0.46	2.67±0.5	4.98	0.000
	Control group	2.18±0.87	2.03±0.81		
Fibrinogen (mg/dl)	Experimental group	275.07±16.8	274.28±33.36	-0.04	0.97
	Control group	243.11±21.53	242.66±19.08		

**Table 3.** The results of Kolmogorov Smirnov test

Variables	Groups	Z	*P*
Age	Experimental group	0.56	0.91
	Control group	0.84	0.47
height (m)	Experimental group	0.4	0.99
	Control group	0.44	0.99
weight (kg)	Experimental group	0.5	0.96
	Control group	0.55	0.92
BMI (kg/m^2^)	Experimental group	0.63	0.81
	Control group	0.85	0.46
PBF (%)	Experimental group	0.63	0.82
	Control group	0.94	0.33
WHR	Experimental group	0.48	0.97
	Control group	0.47	0.98
VFA (cm^2^)	Experimental group	0.64	0.79
	Control group	0.85	0.46
Vo_2_max (l/min)	Experimental group	0.49	0.96
	Control group	0.56	0.9
Homocysteine (mmol/l)	Experimental group	0.66	0.77
	Control group	0.58	0.89
hsCRP (mg/l)	Experimental group	0.54	0.93
	Control group	0.74	0.64
Fibrinogen (mg/dl)	Experimental group	0.54	0.93
	Control group	0.52	0.95

## Discussion

This study was the first study to examine the acute effects of a single bout circuit of resistance exercise with intensity of 35% 1RM on healthy and inactive Iranian middle aged men.

The results of this study showed significant elevation of Hcy after circuit resistance exercise. There is no study for examining the acute effects of circuit resistance exercise on Hcy levels of healthy and inactive middle aged men (14) and the studies about the acute effects of exercise on Hcy levels are different in protocol and subjects from this study. For example, after a competitive marathon race, blood Hcy concentrations raised significantly in twenty two male novice runners (15).

Wright *et al* (1998) showed that thirty minutes cycling with 70% of maximum heart rate did not alter the levels of Hcy in active males (16). Gaume *et al* (2005) reported that a VO_2_max test on a cycle ergometer decreased Hcy levels in 12 male athletes (17). 

Joubert (2008) noted that factors influencing blood concentrations of Hcy are duration, mode and intensity of exercise training and at least sixty minutes exercise with high intensity should be performed to affect Hcy levels (14), but in this study duration of exercise protocol was twelve minutes. Thus it seems that the age of the subjects and the levels of their daily physical activity are other effective factors on serum Hcy levels.

Several mechanisms are responsible for the elevation of Hcy concentrations after the rigorous exercise. High intensity exercise increases the needs of creatine for energy production. Creatine is produced in the liver during the demethylation of methionine and its conversion to Hcy. Thus, creatine production is parallel to Hcy elevation in the body (14). In addition, vitamin B_6_ is a coenzyme for action of transaminases, decarboxylases and glycogen phosphorylase in metabolic pathways of energy production. Consequently, there are not enough vitamins B_6_ in the body for reducing Hcy levels by its conversion to cysteine in transsulfuration pathway. This process leads to Hcy elevation in the blood (14). 

Another finding of this study was a significant elevation of hs-CRP after study protocol. Only two studies examined the effects of exercise training on hs-CRP levels in healthy and sedentary subjects, but both were different from this study in their protocols and subjects. 

Markovitch *et al* (2008) reported no significant changes in hs-CRP levels after thirty minutes treadmill walking at the intensity of 50% VO_2_max and incline of 3% in twelve healthy sedentary males aged from fifty to fifty eight years (11). 

Murtagh *et al* (2005) showed no significant response of CRP following forty five minutes walking program with the intensity of sixty to seventy percents of heart rate maximum in overweight, healthy and inactive men (2). 

Intense muscular contraction and exercise induced muscle injury are the stimulators of IL-6 secretion from muscles. Then IL-6 induces the hepatic synthesis of CRP and its release to the blood stream (8). This mechanism is responsible for the raising of CRP levels after circuit resistance exercise.

In this study, fibrinogen level did not significantly change after the exercise program. No study has been found about the acute response of fibrinogen to single bout of exercise training. IL-6 that stimulates the liver to synthesize CRP also is a stimulator of fibrinogen synthesis by the hepatocytes. Thus, it seems that in response to intense exercise, under induction of IL-6, first blood levels of CRP raise and then fibrinogen levels in the blood stream elevate because in this study hs-CRP level raised significantly, but there was no meaningful change in plasma levels of fibrinogen. Therefore, it is possible that duration and intensity of this training protocol were not enough to affect fibrinogen levels.

## Conclusion

After a single bout of circuit resistance exercise in healthy sedentary middle aged men, serum levels of Hcy and hs-CRP elevate significantly, but plasma levels of fibrinogen do not alter meaningfully. During the exercise, the elevation of creatine synthesis for ATP production increases homocysteine levels (14). Furthermore, muscle-derived IL-6 (a stimulator of glycogenolysis in the liver) induces the hepatic production of CRP (8). It seems that intensity and duration of exercise training to have an effect on blood levels of fibrinogen should be higher than that related to Hcy and hs-CRP. Pathological or beneficial consequences of these changes are not clearly specified. Furthermore, more research is needed to show the acute and chronic effects of physical activity on novel cardiovascular risk factors.
